# Camouflaged Nanosilver with Excitation Wavelength Dependent High Quantum Yield for Targeted Theranostic

**DOI:** 10.1038/s41598-018-34843-4

**Published:** 2018-11-07

**Authors:** Agnishwar Girigoswami, Wafic Yassine, Palani Sharmiladevi, Viswanathan Haribabu, Koyeli Girigoswami

**Affiliations:** Faculty of Allied Health Sciences, Chettinad Hospital and Research Institute (CHRI), Chettinad Academy of Research & Education (CARE), Kelambakkam, Chennai, 603 103 India

## Abstract

The present study shows the thorough investigations on optical properties and hydrodynamic diameters of glutathione (GSH) stabilized nanosilver clusters (AgNC) at different stages of synthesis and engineering for the optimized absolute quantum yield to generate fluorescent images of Dalton Lymphoma Ascites (DLA) tumour bearing mice. The initial increment of quantum yield was wavelength dependent and finally it became 0.509 which was due to the camouflaging or entrapment of AgNC in macrophages membranes. The potentiality of macrophages membrane camouflaged silver nanoclusters (AgM) was reflected in the cell viability assay and confocal based live dead cell assay where the AgM has better cell killing effect compared to AgNC with reduced dosage and *in vivo* mice imaging generated the clear visualization at the tumour sites. Therefore, from the present study, it can be considered that the camouflaged nanosilver can be used for targeted theranostic applications.

## Introduction

Cancer screening can have several issues such as false positives that are critical. Moreover, even with correct diagnoses, the issue arises with treatment of the disease. Traditional chemotherapy has several drawbacks and also depends on the stage at which the cancer is detected. Apart from the treatment related side effects itself, a variety of gastrointestinal, musculoskeletal or constitutional symptoms such as nausea, vomiting, loss of appetite, constipation or diarrhea, fever and fatigue are well reported. Majority of anti-cancer drugs used are hydrophobic in nature which results in poor uptake and circulation of the drug often require a high dose, risking further side effects development. Also considering that most of the drugs used in treatment rely on passive targeting; their toxicity becomes another issue when considering traditional treatments^1^.

Recently, nanotechnology is trying to bridge the gap to overcome most of these drawbacks with an active targeting mechanism, considerably reducing toxicity, increasing specific cancer targeting and cellular uptake. This provides a hydrophilic nature to the drug with the aid of smart nanoformulations, reducing the amount of drug required, improving circulation as well as cellular uptake and providing a cost effective method since anti-cancer drugs tend to be expensive^2,3^.

Silver nanoparticles (AgNPs) are known to have anti-cancer properties, along with a targeting moiety, they can prove to be a good anti-cancer drug by itself^4^. Several active targeting methods are popular for cancer targeting with bio mimicking being a recent popularity due to its stealth properties^5,6^. One of these bio mimicking-inspired targeting methods include using membrane rafts obtained from blood cells with specific interest for the use of macrophage membranes since it contains a receptor known as α4β1 integrin that specifically binds to vascular cell adhesion molecule-1 (VCAM-1)^7,8^. In general, these VCAM-1 are over expressed in cancer cells especially of that in renal cell carcinoma (RCC). Therefore these macrophage rafts can be used to coat the AgNPs to not only give it bio-mimicking stealth properties but also active targeting capability specific to cancer cells^8^. This provides favorable conditions for the use of AgNPs as a therapeutic agent. These AgNPs can further be used for diagnosis as well if they were to have an imaging moiety along with them. It has been shown that silver nanoclusters (AgNCs) have fluorescent properties, if synthesized in a controlled fashion. This tunable fluorescent nature make them superior in comparison to most fluorescent organic dyes in terms of fluorescence intensity, lifetime and toxicity^9^. In this study, an attempt was made to understand the potential theranostic ability of AgNCs with macrophage raft (membrane) coating from blood cells.

## Results and Discussion

### X-Ray Diffraction study

**Figure 1:**
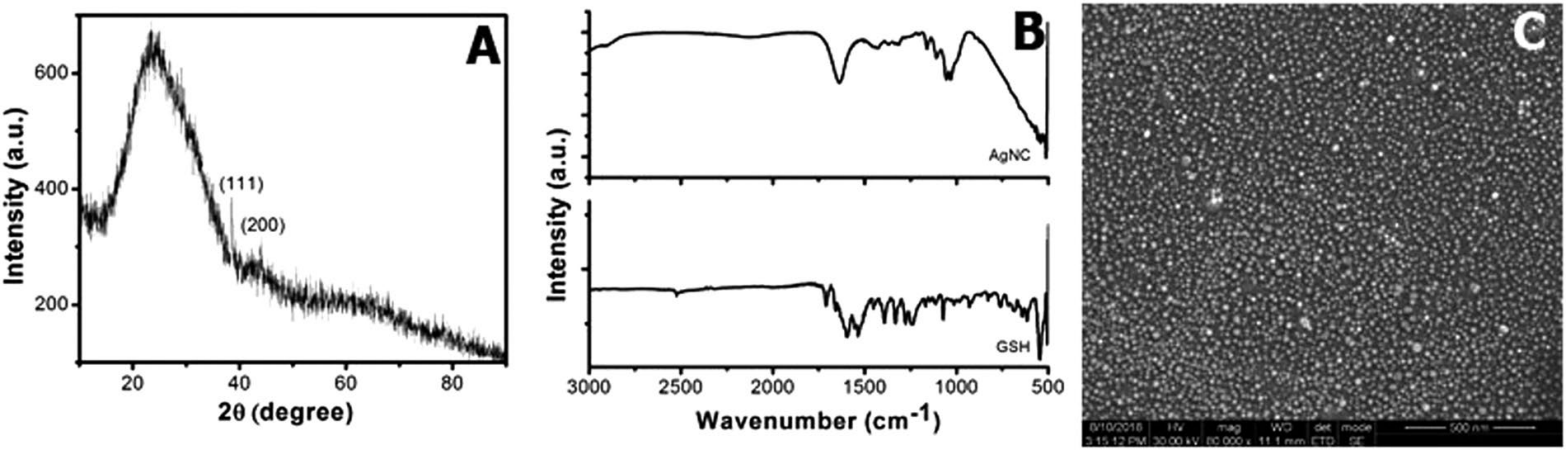
(**A**) X-Ray Diffraction pattern of AgNC. (**B**) FTIR spectra of AgNC and glutathione. (**C**) SEM image of AgNC.

The X-ray diffraction patterns of AgNC shown in Fig. 1(A) exhibit characteristic peaks at 2θ = 38.545° which is attributed to the peak position of silver (111) lattice. The Bragg’s equation yields an average lattice spacing of 0.2334 nm which is very similar to the inter-planar spacing of Ag (111). Another very low peak at 2θ = 44.04° represents the existence of (200) plane. All the characteristic peaks of Ag are very less broadened due to the presence of GSH on the surface of AgNC. The size of the AgNC was estimated applying the Debye-Scherrer equation on the X-ray diffraction peak of (111) plane. The 0.154060 nm wavelength of Cu-K radiation and 11° FWHM were employed to calculate the approximate 0.77 nm diameter of AgNC. X-ray diffraction pattern and the subsequent results are well in agreement with the size obtained from the Scanning Electron Microscopic image of AgNC shown in Fig. 1(C).

### FTIR Analysis

Figure 1(B) represents the Fourier Transform Infrared (FTIR) Spectra taken in ATR mode for GSH and AgNC to confirm the surface modification of silver nanocrystals by GSH. The major IR bands present in the range of 1360–1620 cm^−1^ can be attributed to the symmetric and asymmetric stretching of –COOH functional groups of cysteine. The –S-H stretching vibrations of pure GSH at 2526 cm^−1^ disappeared in the spectrum of AgNC. This result indicates the covalent linking of glutathione on the surface of the silver nanocrystals and the shifting of –COOH frequency in AgNC spectrum took place due to the change in the dipole moment supporting the same.

### Spectrophotometric Assessments

**Figure 2:**
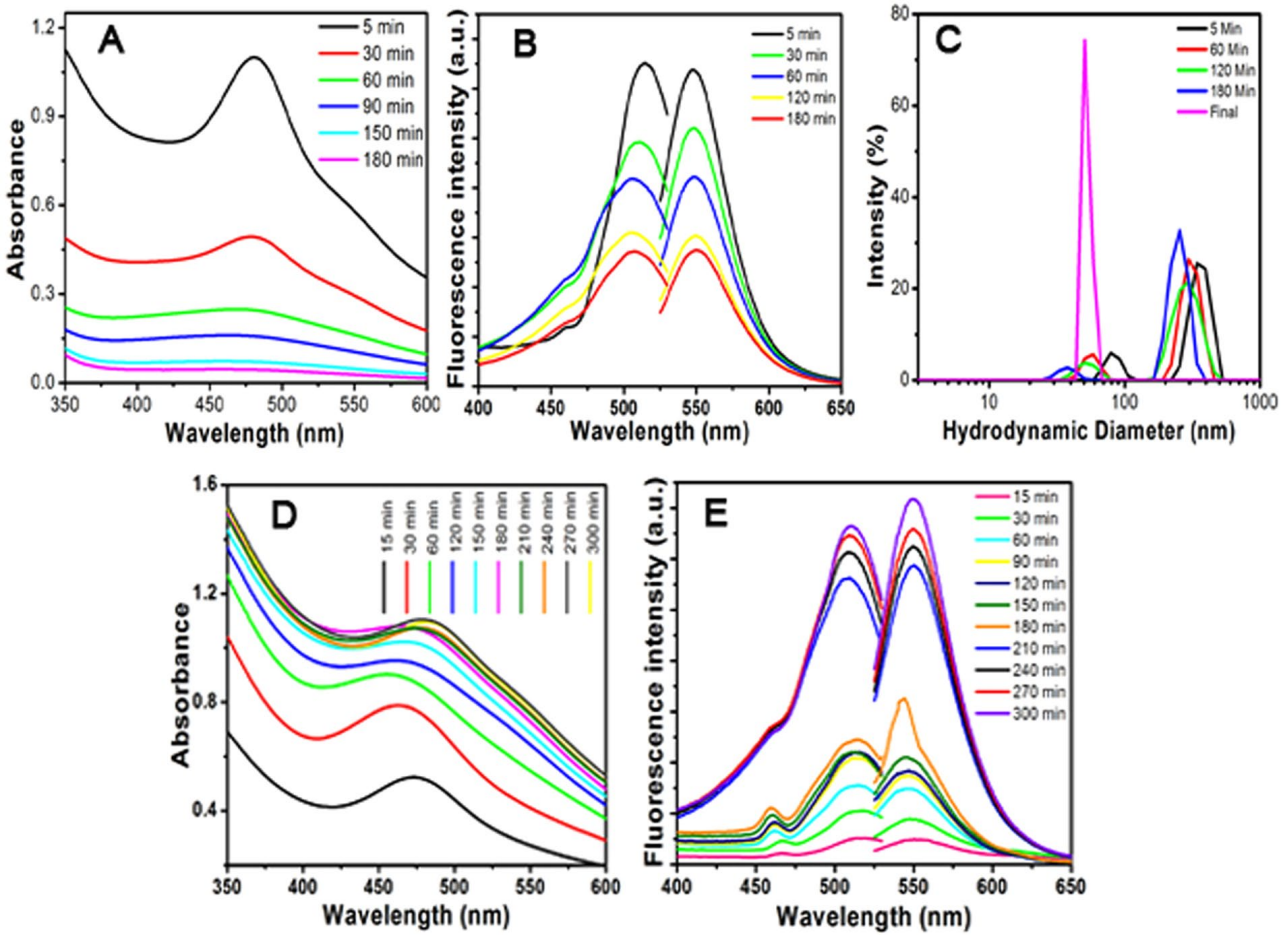
(**A**) Change in the absorption spectra with the time after 1^st^ addition of GSH. (**B**) Emission and excitation spectra after 1^st^ addition of GSH. (**C**) Particle size distribution of AgNC. (**D**) Absorption spectra of AgNC after 2^nd^ addition of GSH. (**E**) Steady-state fluorescence emission and excitation spectra of AgNC after 2^nd^ addition of GSH.

A continuous steady-state spectrophotometric observation was recorded as shown in Fig. 2(A) during the size controlled synthesis of AgNC. An initial addition of alkaline borohydride to the reduced GSH containing AgNO_3_ solution generated brown colour with an absorption maxima at 482 nm to confirm the formation of AgNC. This brown colour started to achromatize with time and this change was reflected in the absorption spectra. The absorption spectra became broad and flat with an increased blue shift in the absorption maxima. The absorption maxima shifted to 466 nm after three hours with 27.5 fold reduction in the absorbance. Further addition of same amount of alkaline borohydride to the mixture resulted in the reappearance of brown colour and there was a drastic change in the absorption spectra as depicted in Fig. 2(D). The absorbance started to increase with a red shift in the absorption maxima after an initial hypsochromic shift till the first hour and maxima became stable after 180 min at 482 nm. After 180 min, the absorbance at 482 nm increased till the 6^th^ hour of incubation and became stable to be stored at 4 °C.

Figure 2(B and E) represents the Time-bound steady-state fluorescence spectra monitored along with the absorption spectra upon exciting at the λ_max_. The wavelength independent λ_em_was observed at 548 nm immediately after first addition of alkaline borohydride and the intensity started to reduce with the time as observed in the absorption spectra. The emission band at 548 nm is a wavelength independent phenomenon for the case of AgNC and emission intensity reached 1/3^rd^ after 180 min. Further addition of borohydride brought change in the emission intensity and started to increase regularly till 180 min. There was a sudden and tremendous increase in the emission intensity after 180 min and at 300 min the intensity became stable.

This fact is attributed to the cyclic reduction-decomposition process of silver thiolates in the presence of alkaline borohydride. NaBH_4_ is a strong reducing agent that reduces Ag^+^ to AgNC in the presence of stabilizing agent, alkaline GSH. The excess thiols then reform silver thiolates through the decomposition process in the inhomogeneous reaction mixture leaving stable silver nanoclusters undisturbed. After second addition of alkaline borohydride to the inhomogeneous reaction mixture the red brown colour is regained indicating the formation of highly luminescent AgNC stabilized by GSH. In the alkaline medium, the -COOH moieties of GSH remain negatively charged and that interacts with the positive surface charge of the metal clusters to stabilize the luminescent AgNC.

Fluorescence excitation spectra were collected by measuring the intensity at λ_em_ maxima (548 nm) over a span of 400–535 nm excitation wavelengths. The maximum intensity was observed at 515 nm with the similar trend as observed in emission spectra for both the additions of borohydride. The trends and nature of the excitation spectra were also similar as was observed in absorption except a 31 nm spectral shift to 515 nm. The excitation spectra overlap more with the emission spectra than the absorption spectra indicating the excitation wavelength dependence of AgNC quantum yield. The similar observation was confirmed by exciting the AgNC at 515 nm. The emission intensity became almost 1.5 fold more at 548 nm compared to the intensity obtained upon exciting the same at its λ_max_ = 482 nm. Absolute fluorescence quantum yields (ϕ) of AgNC were determined using the rhodamine-6G in ethanol as standard. The quantum yields were 0.248 and 0.331 for the excitation wavelength 482 nm and 515 nm respectively. Therefore, it can be concluded that the quantum yield of AgNC is wavelength dependent. For further confirmation, phantom imaging was done using *in vivo* imaging system applying variable wavelength.

### Colloidal Size of AgNC

The colloidal nature of the AgNC was evaluated employing dynamic light scattering measurement. The hydrodynamic diameter was recorded with one hour time interval after addition of alkaline borohydride to resolve on the approximate size of AgNC in mixture. Figure 2(C) shows the recorded changes in hydrodynamic diameter of AgNC at different time intervals. The first datum collected after 5 min of addition, showed a major scattering peak at 364 nm with a minor scattering at 79 nm. There was a continuous shift in both the peaks to the lower hydrodynamic size with the time and after 3 h the major scattering peak was observed at 256 nm with a minor peak at 38 nm. This commensurate to the large thiolate complexes with a very less amount of stable AgNC core that was previously explained by spectrophotometric observation. After 2^nd^ addition of the borohydride, the solution was kept stirring for 15 minutes and allowed to stand for 6 h. The brown color was developed immediately after addition and the color became more intense with the time. The particles size distribution was measured after six hours showing monodispersed distribution at 50.5 nm (final) with 0.04 polydispersity index (PDI). The said peak at 50.5 nm with very high intensity was in the range of minor peak obtained between 79–38 nm after the initial addition of borohydride. The observed result supports the breakdown of Ag-thiolated complex and the formation of AgNC in the same range of initial size in presence of alkaline borohydride and GSH.

### Camouflaged AgNC

**Figure 3:**
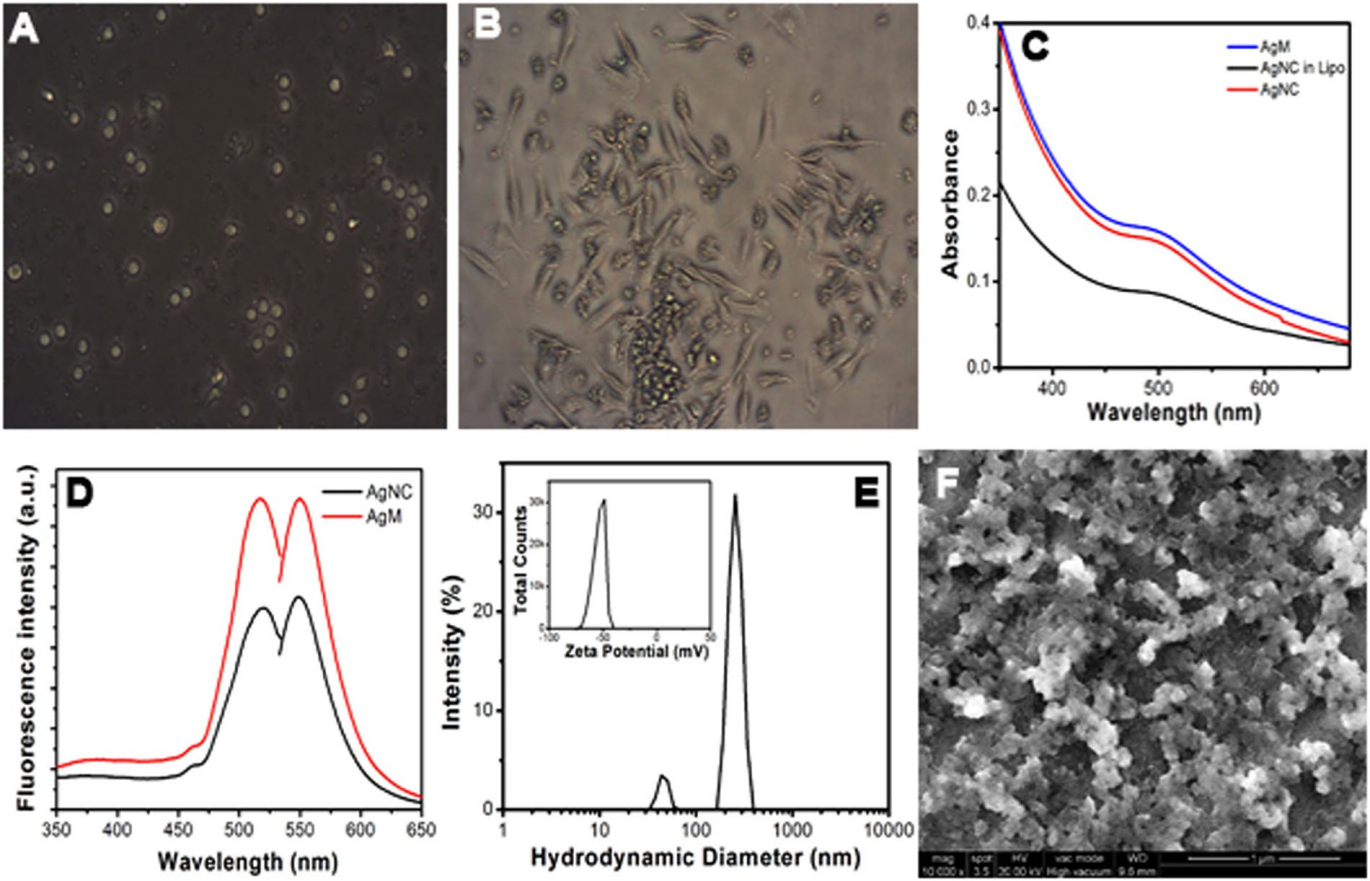
(**A**) Optical microscopic image of isolated white blood cells dispersed in complete medium at day zero. (**B**) At the day 7 showing the transformation of macrophages. (**C**) Absorption spectra of AgNC, liposomal AgNC and AgM. (**D**) Excitation (left) and emission (right) spectra of AgNC and AgM. (**E**) Size distribution graph of AgM and inset shows the zeta-potential of AgM. (**F**) Scanning Electron Microscopic image of AgM.

Macrophages were grown in the complete medium through the differentiation of adherent monocytes shown in Fig. 3(A,B) and after seven days macrophage membranes were isolated. The isolated macrophage membranes were mixed with the AgNC maintaining 1:1 concentration to encapsulate the nanocrystals for targeted delivery to the cancer cells. Membrane encapsulated AgNC showed increment in absorbance with an 18 nm peak shift compared to AgNC (Fig. 3). The emission intensity increased 1.47 times in membrane encapsulated form compared to the AgNC at the λ_em_ maxima 548 nm upon excitation at 515 nm. Proportionate increment in intensity was also observed at 515 nm in the fluorescence excitation spectra. The hypothesis was that the liposomal structure or environment can be generated by the macrophage membrane since it is made mainly of phospholipids. Therefore, the AgNC were incubated with macrophage membrane under constant sonication following the protocol mentioned in our previous work^10^. The increment in absorbance with a red-shift and fluorescence emission was observed after exciting the AgNC at 515 nm. Further, AgNC were taken in the glycerol-water mixture with increasing concentration of glycerol to establish the encapsulation. The behavior of AgNC in water-glycerol mixtures were similar to that observed in macrophage membrane encapsulated forms. Figure 3(C) shows the spectrophotometric observations of AgNC in water-glycerol mixture and that represents the viscosity and polarity driven elevation of optical density and emission intensity confirming the encapsulation. Figure 3(D) shows the emission intensity of AgNC and AgM after exciting both at 515 nm. The absolute quantum yield for AgM was calculated based on the enhanced fluorescence emission intensity at 548 nm compared to AgNC taking rhodamine 6 G as reference that was found to be 0.509. The initial increment of quantum yield was excitation wavelength dependent and the second one for the camouflaging with macrophage membranes.

Figure 3(E) shows the hydrodynamic diameter of membrane encapsulated AgM which was found to be 256.5 nm with 0.13 PDI. The PDI value indicates the monodispersity of the prepared AgM whereas the zeta potential value of −58.9 mV confirms the stability that was −22.3 mV for AgNC earlier. The Fig. 3(F) shows the SEM image of membrane encapsulated AgNC and it has clear visual evidence of membrane encapsulation on AgNC.

### Cytotoxicity assessment

The cytotoxicity assessment of AgNC and AgM was done using MTT assay and the assay showed the dose dependant response on DLA cells (Figure S1). The noticeable cell damage was observed with increasing concentration of both the AgNC and AgM. The LD50 for the AgNC was 12.85 µg/ml whereas it was 8.22 µg/ml for AgM. Most of the cells incubated with AgM at 10 µg/ml concentration were disintegrated as shown in Figure S1(C) whereas, comparatively less cell killing was observed in case of those cells incubated with AgNC (Fig. S1B) at the same concentration of AgNC. These results commensurate the MTT assay results shown in Figure S1(D) indicating the superior cell killing effect of the prepared AgM due to targeting effect that generated from the macrophage membrane encapsulation.

**Figure 4:**
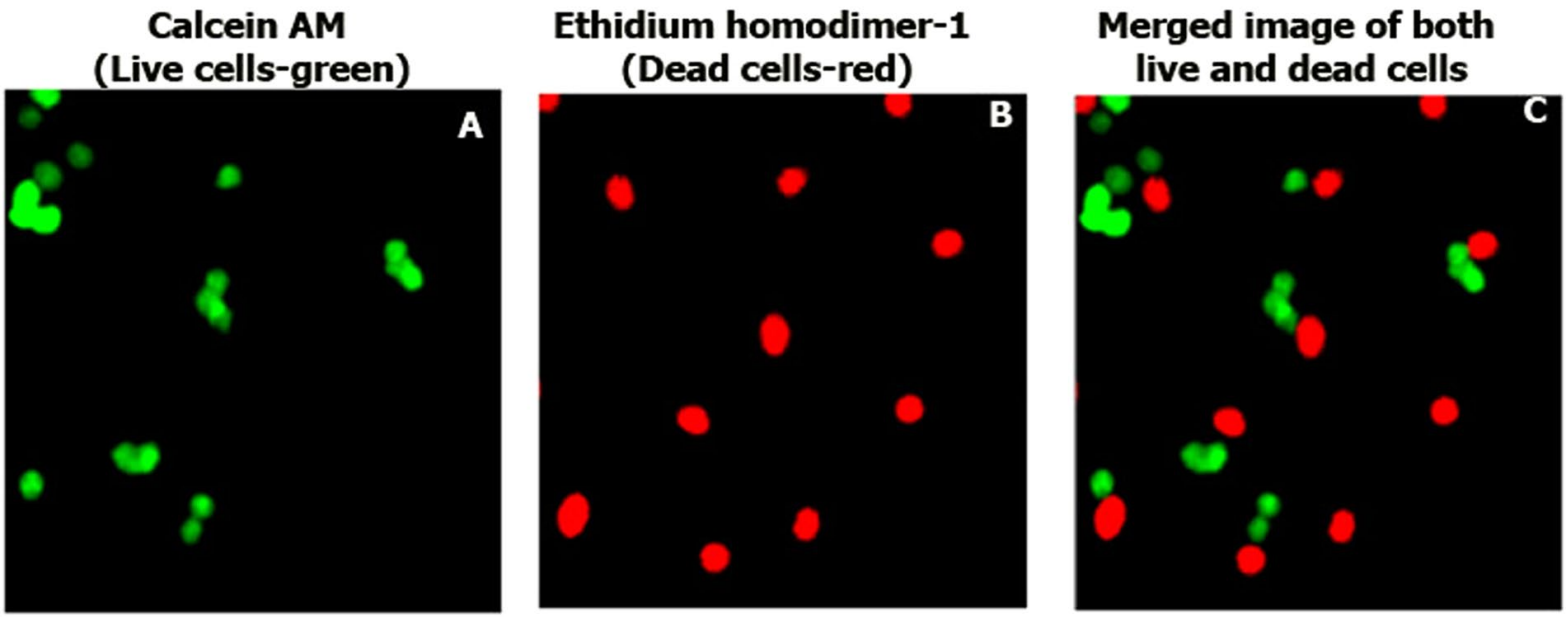
Confocal microscopic images (400× magnification) of DLA cells treated with 7 μg/ml of AgM using Live dead assay kit. (**A**) Live cells with green fluorescence (calcein- AM), (**B**) Dead cells with red fluorescence (ethidium homodimer-1) and (**C**) Both live and dead cells in merged image.

To further confirm the toxic capacity of the AgM over DLA cells we have used calcein AM and ethidium homodimer-1 dyes to stain the live (green) and dead (red) cells using fluorescent colours respectively, which was visualized using confocal microscopy (Fig. 4).

### *In vitro* and *in vivo* fluorescence imaging

**Figure 5:**
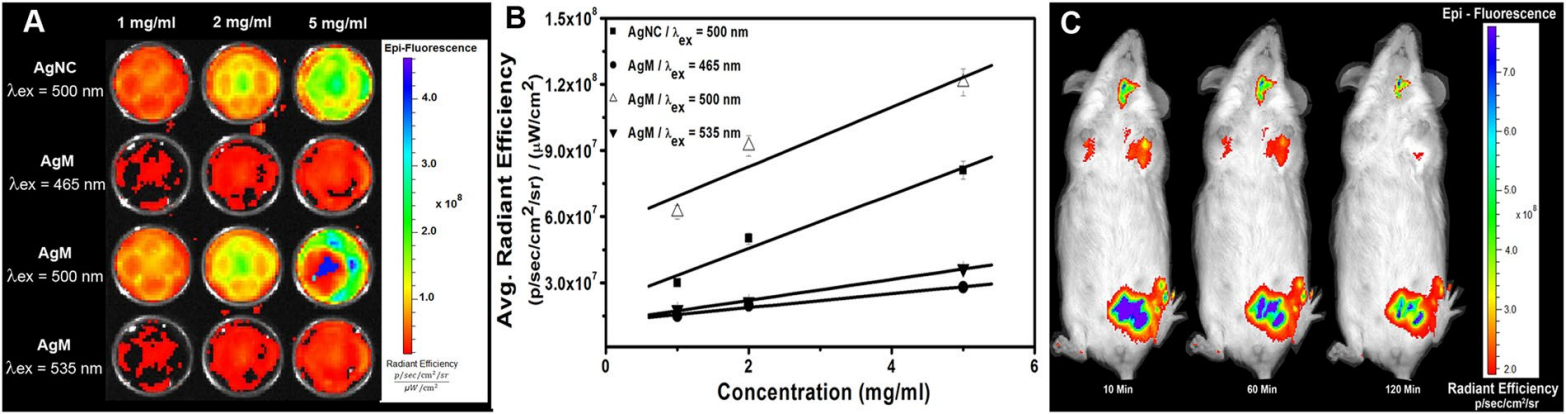
(**A**) Phantom fluorescent images of variable concentration of AgNC and AgM at different λ_ex_. (**B**) Showing the changes of Average Radiant Efficiency against the variable concentrations of AgM and AgNC at different λ_ex_. (**C**) *In vivo* fluorescence images of tumor bearing mouse with AgM injected intravenously at different time interval.

Phantom (*in vitro*) fluorescence imaging studies were performed using IVIS Lumina *in vivo* imaging system after taking varying concentrations of AgM in 24 wells plate. The Fig. 5(A) shows the phantom images after exciting the sample at three different wavelengths keeping the emission wavelength fixed at 550 nm. All the fluorescence emissions were normalized to photons per second per centimeter per steradian (p/sec/cm^2^/sr) after deducting the background signals. The AgM shows linear increment in the intensity with increasing concentrations at 500 nm excitation wavelength whereas excitation at 465 nm and 535 nm generated comparatively weak signals. Figure 5(B) shows the graphical representation of average radiant efficiency with the corresponding nanoclusters concentrations. If the slope is considered to represent the average efficiency, then the average radiant efficiency for AgM at 500 nm excitation would be 1.35 × 10^7^ p/sec/cm^2^/sr per mg/ml which was higher than the AgNC (1.22 × 10^7^ p/sec/cm^2^/sr per mg/ml) at same wavelength of excitation. This increment is attributed to the camouflaging of AgNC in liposome like structures that was formed by the macrophage membranes. This experiment also confirms the wavelength dependence of quantum yield.

In order to test the targeted imaging potential of AgM, the particles were intravenously injected to DLA tumour bearing mice and imaged with regular intervals. The maximum intensity was observed after 30 minutes post injection at the peritoneal cavity where the DLA tumour exists. The image was normalized on the same scale of p/sec/cm^2^/sr. The image intensity started to fade after 1 h and it became faint after 2 h as shown in the Fig. 5(C). This fact illustrates that the macrophage membrane coatings over the nanocrystals help the particles to attach on the tumour cells through the membrane bound proteins. This may be the classical example of the active targeting ability of camouflaged AgM after recognizing the tumour endothelium that helps accumulation of the fluorescent particles around the tumour cells.

In conclusion, the synthesis of AgNC was confirmed by X-ray diffraction and FTIR analysis. The specific crystal properties and characteristic bands were respectively identified. Further, it was reasoned that nanosilver clusters (AgNC) can be synthesized with variable optical properties at each step by forming Ag-thiol complexes in the presence of GSH which was depicted by the changes in spectrophotometric observations as discussed. The change in the fluorescence spectra and intensities of AgNC at each step of NaBH_4_ addition indicated that the luminescent properties of the AgNC are dependent on the wavelength at which they were excited. A step forward, when the AgNC is camouflaged with macrophage membranes (AgM) forming similar formulation as that with liposomes, they show better increase in absorbance and fluorescence intensity with a hold on their wavelength dependence as that of AgNC before encapsulation. The phantom fluorescence imaging represents the increased quantum yields of AgM which is further substantiated with the targeted accumulation and fluorescence from the tumor site *in vivo*. Cytotoxicity assessments on the other hand confirmed the cell-killing effect of these AgM which is superior at a lower dose in comparison with the AgNC on DLA tumour cells. It is therefore complementing that Nanosilver clusters camouflaged with macrophage membranes could be a better targeted nano-formulation that can be used as a theranostic agent in fluorescence imaging due to the wavelength dependence of silver nanoclusters as well as therapeutic agent for cancer due to its cell killing effects.

## Methods

### Synthesis of AgNCs

125 μl of 20 mM AgNO_3_ was prepared in deionized water (DW) to which 150 μl of 50 mM GSH was added with continuous stirring and the volume was made up to 15.275 ml to produce a white Ag-thiol complex. 112 mM NaBH_4_ in 8 ml along with 2 ml of 1 M NaOH was prepared and from which 150 μl was added to Ag-thiol complex. This produced a deep red solution and was stirred for 5 min followed by collection of 5 ml of the above solution and aging for 3 h to obtain a colorless solution. 250 μl NaBH_4_ was added to the faded solution which produced a pale brown solution after 15 min of stirring and was left overnight at room temperature without disturbance and the sample was stored at 4 °C for further use.

### Culture and isolation of macrophage membrane

5 ml peripheral blood was drawn and mixed with equal volumes of PBS containing 10 mg of EDTA. This was layered over 3 ml of ficoll - 1077 followed by centrifugation at 400 g for 20 min. The white buffy coat layer was carefully collected and after washing several times in 4 ml phosphate buffer saline (PBS) the pellet was dispersed in Dulbecco’s modified Eagle medium (DMEM) with 10% fetal bovine serum (FBS) and 1% antibiotic solution. Thus isolated cells were then left in CO_2_ incubator for 4 days. On the fourth day, adherent mononuclear cells were left in fresh medium after discarding the free floating cells.

### Isolation of plasma membrane rafts

At day 7 of culturing, the macrophages were trypsinized and were pelleted followed by washing in ice cold TM (50 mM Tris Cl, pH 7.5, 10 mM Magnesium Sulfate) buffer for three times. Finally the pellet was dispersed in 4 ml TM buffer and was layered over 2.66 ml of 1 M sucrose solution. This was then centrifuged at 2000 g for 10 minutes at 4 °C. The pellet was dispersed in TM buffer and centrifuged at 3000 g for 30 minutes at 4 °C and the pellet was washed 3 times and finally was dispersed in 4 ml buffer to store at 4 °C for further use.

### Preparation of Macrophage coating over nano silver

The isolated membrane was mixed with equal volume of silver nanocrystals and incubated at room temperature for 1 h. The membrane coated nanocrystals were then used for characterization and consequent experiments.

### MTT assay

The MTT (3-(4, 5-dimethylthiazol-2-yl)-2, 5-diphenyltetrazolium bromide) tetrazolium reduction assay was used to determine cell viability. DLA (Dalton’s Lymphoma Ascites) cells were cultured in a 24 well plate, each well containing 1 ml complete medium (DMEM supplemented with 10% FBS and 1% antibiotics) and cultured along with varying concentration of samples and kept for 24 h after which 100 μl MTT reagent (5 mg/ml) was added in each well and left for 4 h to incubate. After this, 1 ml of Dimethyl sulfoxide (DMSO) was added to each well to dissolve formazan crystals and the optical density was measured at 570 nm^11^.

#### Live dead cell assay

DLA cells were treated with 7 μg/ml of AgM for 24 h and were processed for live dead cell assay using a combination of calcein AM and ethidium homodimer-1 dye which gives green fluorescence for live cells and red fluorescence for dead cells respectively. The staining was done in dark conditions and was imaged in Carl Zeiss laser scanning confocal microscope according to Girigoswami *et al*.^12^. The experiment was repeated thrice.

#### Induction of DLA tumor

The DLA tumor cell line was propagated into transplantable tumor in the peritoneal cavity of *Swiss albino* mice following the approved protocol of CPCSEA affiliated Institutional Animal Ethics Committee (IAEC) of Chettinad Academy of Research & Education (Registration no. 944/ac/06/CPCSEA)^13^. All animal experiments were supervised and approved by the IAEC (IAEC2/Desp. No. 06) of Chettinad Academy of Research & Education, Kelambakkam, Chennai, INDIA.

Shizmadzu (Japan) UV - 1800 spectophotometer and Jasco FP- 8500 spectrofluorometer were used to measure the absorption and steady-state emission spectra for the synthesized nanocrystals. Bruker ALPHA attenuated total reflection Fourier transform infrared spectroscopy (ATR-FTIR) was employed to understand the bonding information of the synthesized clusters. The crystallographic phase of the nanoclusters was determined on Rigaku D/max–2500 diffractometer over the 2θ range from 10–70°. The colloidal nature was examined by Nano-Zs (Malvern) instrument, which is equipped with a 4 mW He-Ne laser (λ = 633 nm). All the scattered photons were collected at 90° scattering angle to process the hydrodynamic diameter and ζ-potential using the Malvern zetasizer software. FEI quanta FEG-200 high resolution scanning electron microscope was used to measure the actual size and formation of the nanoclusters.

## Electronic supplementary material

Dataset 1
