# Supplementary material for: Camouflaged Nanosilver with Excitation Wavelength Dependent High Quantum Yield for Targeted Theranostic
Source: Sci Rep. 2018 Nov 7;8:16459. doi: 10.1038/s41598-018-34843-4 (PMC6220276; doi:10.1038/s41598-018-34843-4)
Supplement: Supplementary file 1 — Dataset 1 [file 41598_2018_34843_MOESM1_ESM.pdf]

Supplementary Information

Camouflaged Nanosilver with Excitation Wavelength Dependent High Quantum Yield for Targeted Theranostic

Agnishwar Girigoswami*, WaficYassine, Palani Sharmiladevi, Viswanathan Haribabu, Koyeli Girigoswami

Faculty of Allied Health Sciences, Chettinad Hospital and Research Institute (CHRI), Chettinad Academy of Research & Education (CARE), Kelambakkam, Chennai 603 103, INDIA

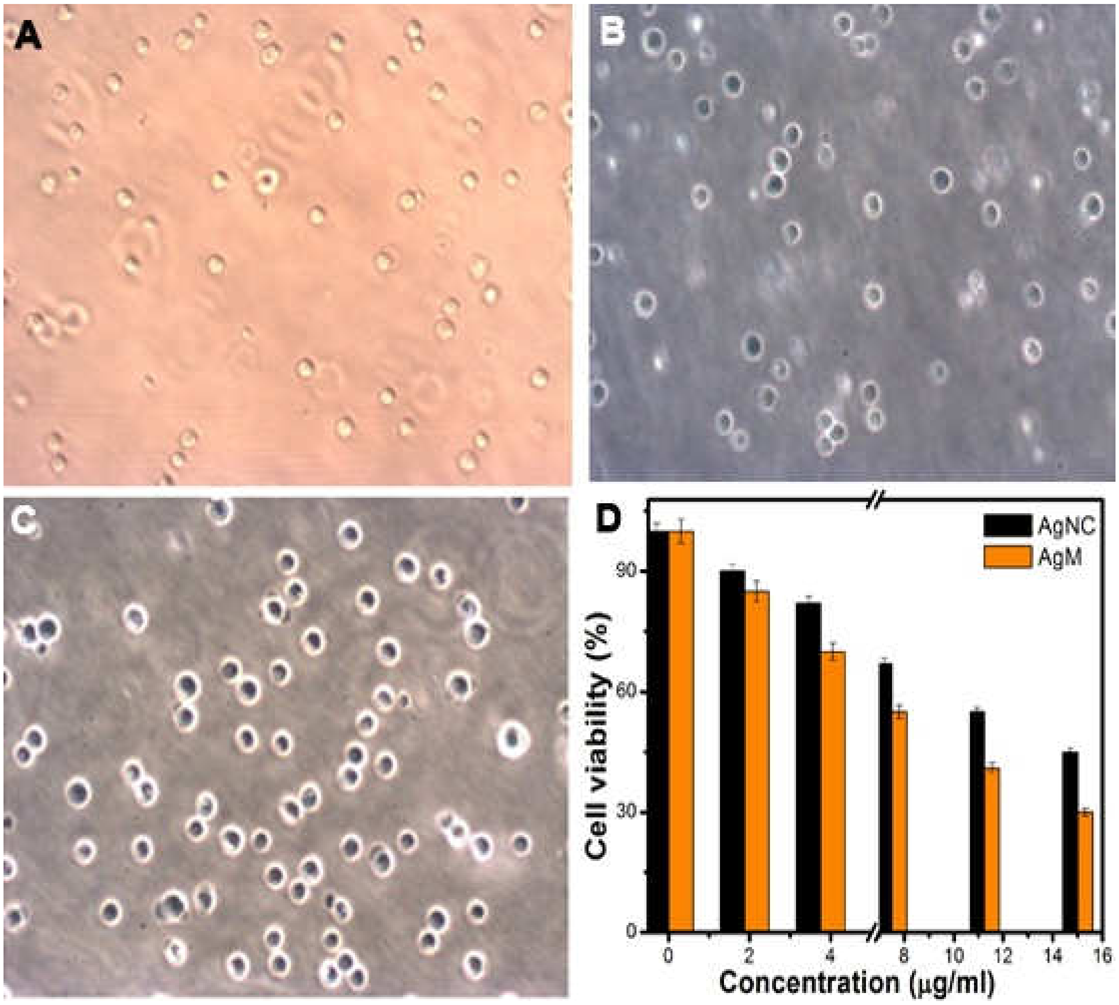

Fig. S1: Optical microscopic image of A. DLA tumor cells, B. Trypan blue stained DLA treated with 10 μg/ml of AgNC, C. Trypan blue stained DLA tumor cells treated with 10 μg/ml AgM. D. Graphical representation of cell viability assay of DLA tumor cells after treating with AgNC and AgM.
